# Association Between Cytomegalovirus Serostatus, Antiviral Therapy, and Allograft Survival in Pediatric Heart Transplantation

**DOI:** 10.3389/ti.2022.10121

**Published:** 2022-03-16

**Authors:** Naveed Rabbani, Richard A. Kronmal, Thor Wagner, Mariska Kemna, Erin L. Albers, Borah Hong, Joshua Friedland-Little, Kathryn Spencer, Yuk M. Law

**Affiliations:** ^1^ Division of Pediatric Cardiology, Seattle Children’s Hospital, Seattle, WA, United States; ^2^ Department of Biostatistics, University of Washington, Seattle, WA, United States; ^3^ Division of Pediatric Infectious Diseases, Seattle Children’s Hospital, Seattle, WA, United States

**Keywords:** heart transplantation, graft survival, infection, antiviral, cytomegalovirus, pediatrics

## Abstract

**Background:** Cytomegalovirus (CMV) is an important complication of heart transplantation and has been associated with graft loss in adults. The data in pediatric transplantation, however, is limited and conflicting. We conducted a large-scale cohort study to better characterize the relationship between CMV serostatus, CMV antiviral use, and graft survival in pediatric heart transplantation.

**Methods:** 4,968 pediatric recipients of solitary heart transplants from the Scientific Registry of Transplant Recipients were stratified into three groups based on donor or recipient seropositivity and antiviral use: CMV seronegative (CMV-) transplants, CMV seropositive (CMV+) transplants without antiviral therapy, and CMV+ transplants with antiviral therapy. The primary endpoint was retransplantation or death.

**Results:** CMV+ transplants without antiviral therapy experienced worse graft survival than CMV+ transplants with antiviral therapy (10-year: 57 vs 65%). CMV+ transplants with antiviral therapy experienced similar survival as CMV- transplants. Compared to CMV seronegativity, CMV seropositivity without antiviral therapy had a hazard ratio of 1.21 (1.07–1.37 95% CI, *p*-value = .003). Amongst CMV+ transplants, antiviral therapy had a hazard ratio of .82 (0.74–.92 95% CI, *p*-value < .001). During the first year after transplantation, these hazard ratios were 1.32 (1.06–1.64 95% CI, *p*-value .014) and .59 (.48–.73 95% CI, *p*-value < .001), respectively.

**Conclusions:** CMV seropositivity is associated with an increased risk of graft loss in pediatric heart transplant recipients, which occurs early after transplantation and may be mitigated by antiviral therapy.

## Graphical Abstract

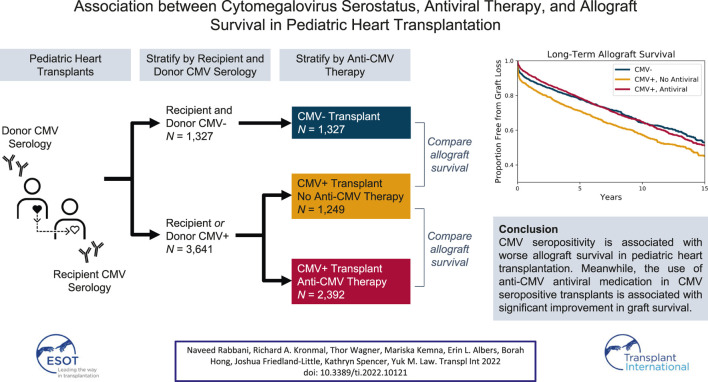

## Introduction

Cytomegalovirus (CMV) infection is a common complication after heart transplantation (1). There is growing evidence that in addition to causing acute illness, CMV infection also contributes to cardiac allograft vasculopathy and long-term graft loss in adult heart transplant recipients (2–6). CMV infection may be associated with poor outcomes in pediatric recipients as well, but the data is limited and conflicting (7–9).

Large, high-quality studies from the 1990s established that anti-CMV treatment following transplantation reduces the risk for acute CMV illness (10) as well as cardiac allograft vasculopathy in adult recipients (11). This has been the main motivation for the use of CMV prophylaxis in heart transplant recipients. However, there is not yet a consensus, particularly in pediatric heart transplantation, regarding which patients should receive post-transplantation antiviral therapy.

Traditionally, risk for acute CMV infection is stratified by donor (D) and recipient (R) serostatus combination, with D+/R− considered to be the highest risk. Thus, these patients were the first to widely receive CMV prophylaxis. However, there is some evidence that anti-CMV therapy may be beneficial in all CMV seropositive transplants, regardless of whether the recipient or donor is positive (12–15).

In order to advance post-transplant antiviral practice in pediatric heart transplant, we sought to better characterize both the impact of CMV serostatus and CMV antiviral therapy on graft survival. We present the findings of a large-scale cohort study using the Scientific Registry of Transplant Recipients (SRTR) to answer these two questions.

## Material and Methods

We performed a cohort study of pediatric heart transplants using de-identified data from the SRTR database. The SRTR data system includes data on all donor, wait-listed candidates, and transplant recipients in the United States, submitted by the members of the Organ Procurement and Transplantation Network (OPTN). The Health Resources and Services Administration (HRSA), U.S. Department of Health and Human Services provides oversight to the activities of the OPTN and SRTR contractors.

Our cohort included patients younger than 21 years of age at time of transplant who underwent solitary primary heart transplantation in the United States between 1987 and March 30, 2015. Follow-up information was available through August 2019 with a median follow-up time of 7 years. The primary outcome for analysis was graft loss, as defined by either death or retransplantation.

**FIGURE 1:**
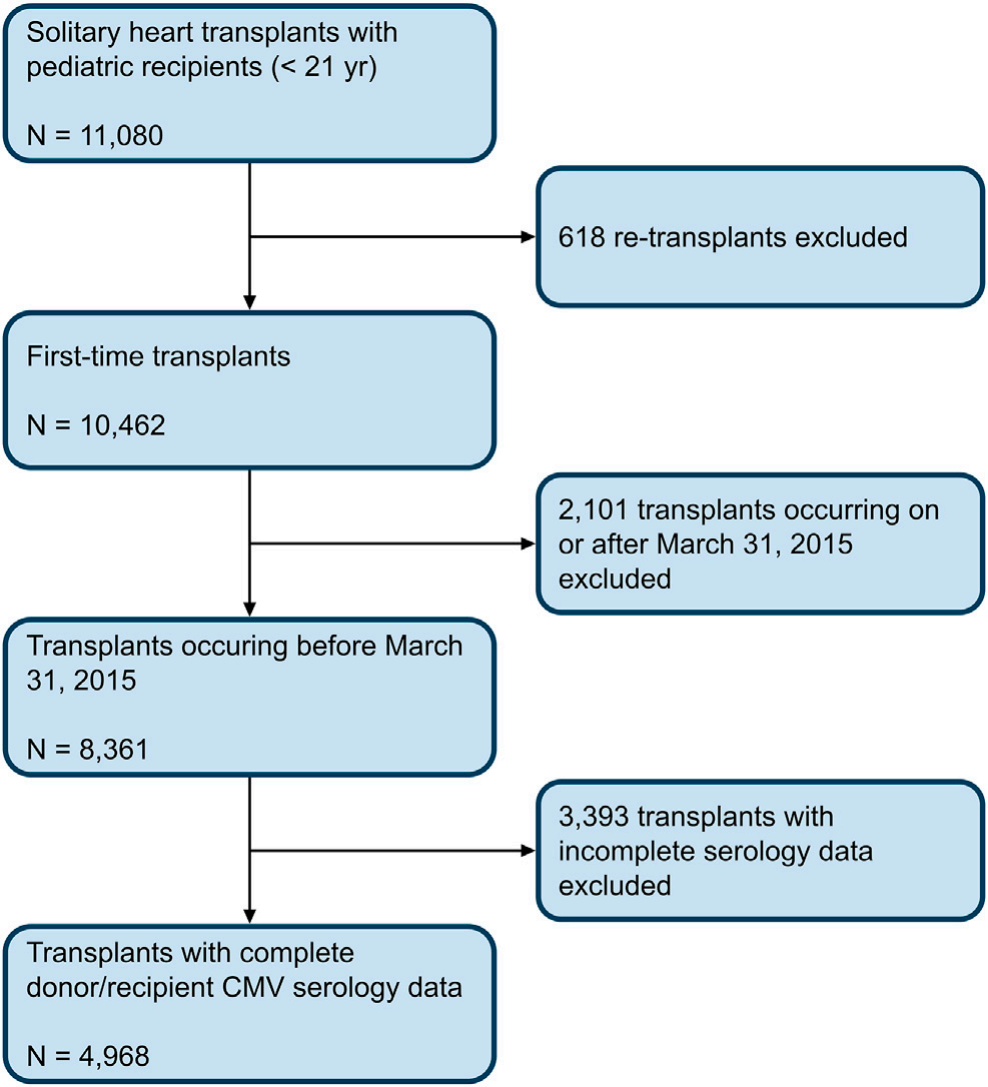
Cohort Selection Criteria. Flowchart depicting the inclusion criteria used to select the cohort of transplants from the 2019 SRTR database.

Transplants occurring on or after March 31, 2015, were excluded, since questions regarding CMV serology and CMV antiviral therapy were no longer included in the SRTR data collection from that point onward. Retransplantation and multi-organ transplants were excluded. Any transplants with missing CMV serology status for donor or recipient were excluded (Figure 1). A transplant was deemed to be CMV serostatus positive if either the donor and/or recipient had positive CMV serologies. A patient was considered to have received CMV antiviral therapy if the registry indicated that the patient received either ganciclovir or valganciclovir after transplantation. An age threshold of 21 years was chosen to match our own clinical practice. At our pediatric institution, we regularly perform heart transplants on young adult patients, many of whom are diagnosed with heart disease as children and cared for accordingly by our pediatric transplant team.

Several covariates, including recipient and donor demographic information and medical history were extracted from the database, and are summarized in the following section of this manuscript. Post-transplant dialysis was specifically included since renal failure after transplantation may be a relative contraindication to antiviral use. Year of transplant was also included to account for era effect. Candidates with adult listing status were converted to an equivalent pediatric status and all pediatric statuses were simplified to status 1 or 2. Recipients were deemed to have congenital heart disease if any of the following fields in the database were marked: valvular heart disease, congenital heart defect, hypoplastic left heart syndrome, congenital heart defect with surgery, or congenital heart defect without surgery. The field for anti-CMV immunoglobulin therapy was sparsely populated, and recipients were assumed to not have received this treatment unless explicitly indicated in the database.

Kaplan-Meier survival models were created to estimate overall graft survival in the entire cohort as well as by donor-recipient CMV serostatus combination groups (D+/R+, D+/R-, D-/R+, D-/R-). Amongst each of these four groups, additional survival models and pairwise log-rank tests were calculated comparing graft survival in recipients who received antiviral therapy to those who did not.

To better characterize the relationship between CMV serology status, antiviral therapy, and graft survival, recipients were then stratified into three groups: recipients of CMV serostatus negative transplants (CMV-, defined as D-/R- transplants), recipients of CMV serostatus positive transplants (CMV+, defined as D+/R+, D+/R-, and D-/R+ transplants) who did not receive antiviral therapy, and recipients of CMV+ transplants who received antiviral therapy. This stratification allows one to separate the effect of CMV positivity and antiviral therapy, which are often confounded. CMV- transplants were not further separated by antiviral use since antiviral therapy was not expected to have an effect on graft survival in these transplants. This assumption is later confirmed by the donor-recipient subgroup analysis and is discussed in further detail in the Results section.

Transplant characteristics and summary statistics were computed across these three groups. Kaplan-Meier survival curves were then calculated comparing graft survival. A multivariable Cox proportional hazards model was created using the trichotomous stratification above in addition to clinically relevant covariates, which were pared down by backwards elimination to include only statistically significant predictors. In order to estimate the effect of untreated CMV seropositivity on graft loss, a hazard ratio was calculated comparing CMV negativity to CMV positivity without antiviral therapy. Additionally, to estimate the effect of antiviral use on graft loss, a hazard ratio was calculated comparing CMV positivity without antiviral therapy to CMV positivity with antiviral therapy.

To further control for potential confounding, the multivariable model above was also recalculated with the addition of a propensity score estimating the probability of antiviral use amongst CMV+ transplants. The propensity score was computed using a logistic regression model whose components were selected from the same pool of clinical co-variates above and pared down via backwards elimination to include only statistically significant predictors.

A second multivariable Cox proportional hazards model was created to estimate the risk of graft loss occurring within the first year after transplant. Covariates were again selected by backwards elimination and a model was created both with and without the antiviral use propensity score. Using the same methodology as above, a hazard ratio was calculated by contrasting CMV- transplants to CMV+ transplants without antiviral therapy and by contrasting CMV+ transplants that did not receive therapy to CMV+ transplants that did. In order to assess for the possibility of selection bias for those who survived the early post-operative period, this 1-year survival model was also recalculated excluding recipients who had a graft loss event within the first week after transplantation.

The dataset was prepared using Python 2.7 with the PANDAS library (version 0.24.2) (16). Summary statistics and Kaplan-Meier survival curves were created using Python 2.7 with the SciPy (version 1.2.0) and LifeLines (version 0.19.5) libraries (17,18). Cox proportional hazards models and propensity scores were computed using STATA 15. *p*-values less than 0.05 were considered statistically significant. The study was approved by Seattle Children’s Institutional Review Board (approval number STUDY00002063, protocol HRP-503B).

## Results

A total of 8,361 patients younger than 21 years of age underwent primary, solitary heart transplantation in the United States between May 25, 1987 and March 30, 2015. Of these, 4,968 had complete CMV serology data available and were included in the final analysis. The median transplant year was 2008, with 4,755 transplants (96%) occurring during or after the year 2000. There were 1,239 D+/R+ transplants, 1,482 D+/R- transplants, 920 D-/R+ transplants, and 1,327 D-/R- transplants. Within these groups, the proportion of CMV antiviral use was 65, 71, 58, and 33% respectively. Of all included transplants, 350 (7%) ended in retransplant and 1,544 (31%) ended in death, for a total of 1,894 (38%) graft loss events.

**FIGURE 2:**
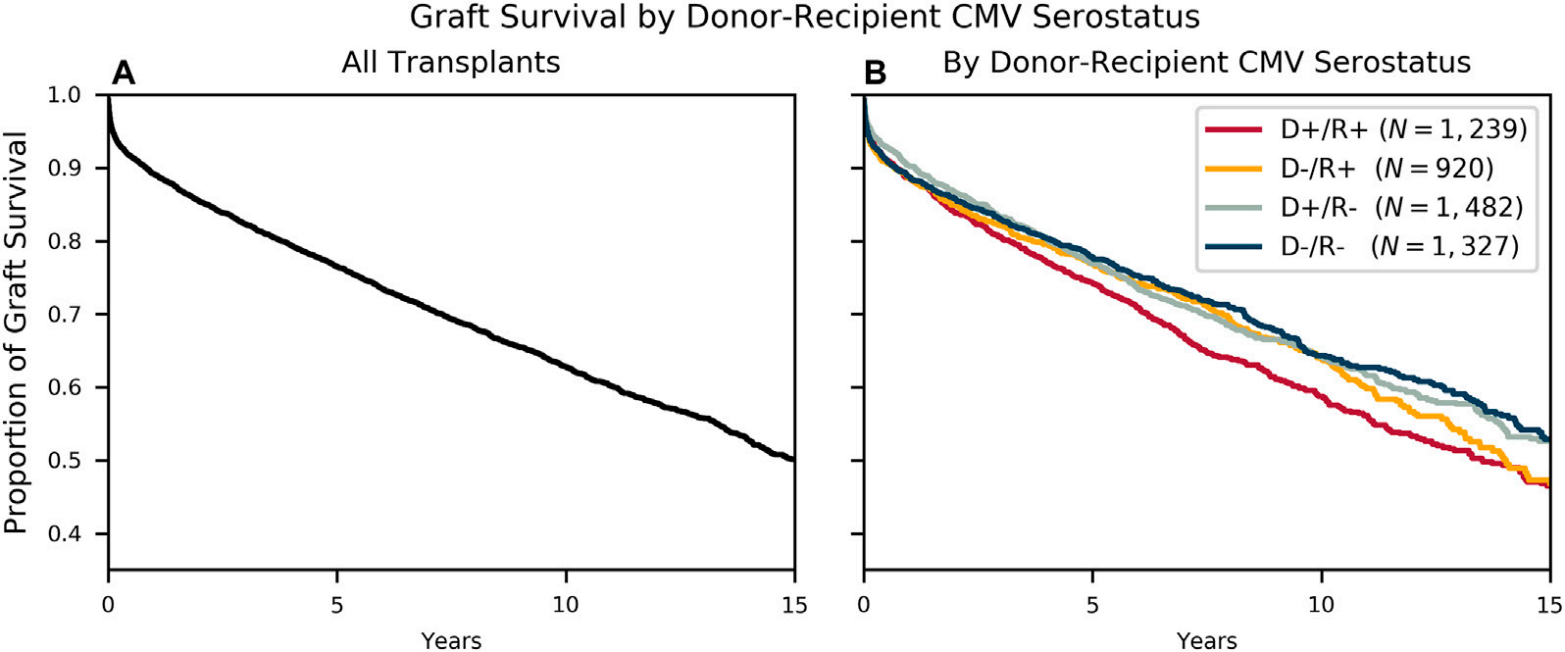
Survival by Donor-Recipient Serostatus. Kaplan-Meier survival curves modeling freedom from allograft loss **(A)** for the entire cohort and **(B)** stratified by the four donor-recipient serostatus combinations.

The overall estimated 10-year cohort graft survival rate was 63%. Subsequent Kaplan-Meier survival models stratified by donor-recipient CMV serostatus showed a 10-year graft survival of 59% for D+/R+ transplants and 64% 10-year survival for the other three groups (Figure 2).

**FIGURE 3:**
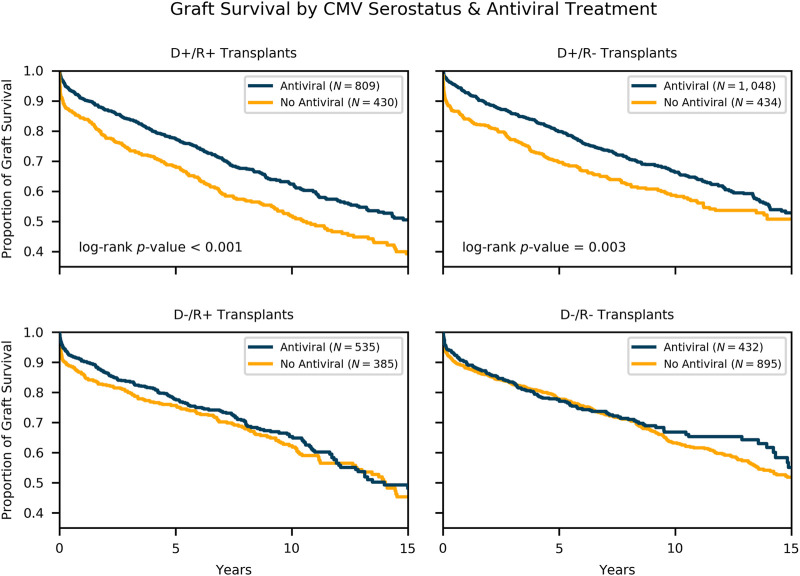
Survival by Donor-Recipient Serostatus and Antiviral Therapy. Kaplan-Meier survival estimates of freedom from allograft loss comparing recipients who received antiviral therapy to those who did not, stratified by donor-recipient serology combination. In this subgroup analysis, antiviral therapy was associated with a statistically significant improved survival in D+/R+ and D+/R- subgroups.

For each of the four CMV serology groups, Kaplan-Meier survival curves were calculated comparing graft survival in those recipients who received CMV antiviral therapy to those who did not (Figure 3). Antiviral therapy was associated with improved freedom from graft loss in D+/R+ transplants (10-year survival of 62 vs 52%, log-rank *p*-value < .001) and D+/R- transplants (10-year survival of 66 vs 59%, log-rank *p*-value = 0.003). The difference in survival was observed early after transplantation and holds throughout the follow up period. For D-/R+ transplants, there is early separation between the curves, however, the log-rank test is not significant. As expected, the D-/R- survival plots showed no appreciable difference between the two treatment groups. Importantly, these survival curves demonstrate that amongst D+ transplants, recipients who received antiviral therapy achieved similar overall graft survival compared to recipients of CMV- transplants.

Further analysis was done comparing CMV- transplants to CMV+ transplants without antiviral therapy and subsequently CMV+ transplants without antiviral therapy to CMV+ transplants with antiviral therapy. This method allows one to separate the effects of CMV serostatus positivity and antiviral therapy in a multivariable risk regression model. These are exposures that are otherwise strongly correlated and confounded. When all eight donor-recipient-antiviral combinations were included in this multivariable model, no additional predictive value was achieved, which is further evidence that the three-group analysis is sufficient to describe the association between CMV serostatus, CMV antiviral therapy, and graft loss.

Table 1 summarizes the donor, recipient, and transplant characteristics that were used to create the adjusted multivariable risk models. There were 1,327 CMV- transplants, 1,249 CMV+ transplants without antiviral therapy, and 2,392 CMV+ transplants with antiviral therapy.

**TABLE 1:** Demographic and clinical characteristics stratified by CMV serostatus and antiviral use.

	CMV-	CMV+, No Antiviral	CMV+, Antiviral	All	N
	*N = 1,327*	*N = 1,249*	*N = 2,392*	*N = 4,968*	
Transplant Outcomes					
Retransplant	85 (6%)	95 (8%)	170 (7%)	350 (7%)	4,968
Death	379 (29%)	471 (38%)	694 (29%)	1,544 (31%)	4,968
Graft Loss (Retransplant or Deazh)	464 (35%)	566 (45%)	864 (36%)	1,894 (38%)	4,968
Transplant Year (median ± i.q.r.)	2009 ± 8 years	2007 ± 8 years	2008 ± 8 years	2008 ± 8 years	4,968
ABO Incompatibility	44 (3%)	33 (3%)	57 (2%)	134 (3%)	4,968
Ischemic Time (min) (mean ± s.d.)	218 ± 71	212 ± 72	217 ± 74	216 ± 73	4,787
Post-Transplant Dialysis	74 (6%)	89 (7%)	145 (6%)	308 (6%)	4,968
D:R Weight Ratio (mean ± s.d.)	1.4 ± 0.5	1.3 ± 0.5	1.3 ± 0.5	1.3 ± 0.5	4,967
D:R Height Ratio (mean ± s.d.)	1.1 ± 0.2	1.1 ± 0.4	1.1 ± 0.2	1.1 ± 0.3	4,944
Recipient Characteristics					
*Age*					4,968
<1 year	392 (30%)	373 (30%)	556 (23%)	1,321 (27%)	
1–3 years	229 (17%)	158 (13%)	256 (11%)	643 (13%)	
3–6 years	143 (11%)	78 (6%)	221 (9%)	442 (9%)	
6–12 years	191 (14%)	195 (16%)	383 (16%)	769 (15%)	
>12 years	372 (28%)	445 (36%)	976 (41%)	1,793 (36%)	
Gender (Male)	742 (56%)	677 (54%)	1,336 (56%)	2,755 (55%)	4,968
*Race*					4,968
White	1,005 (76%)	846 (68%)	1,742 (73%)	3,593 (72%)	
Black	247 (19%)	328 (26%)	476 (20%)	1,051 (21%)	
Other	75 (6%)	75 (6%)	174 (7%)	324 (7%)	
CMV+ Serology Status	0	815 (65%)	1,344 (56%)	2,159 (43%)	4,968
Antiviral Therapy	432 (33%)	0	2,392 (100%)	2,824 (57%)	4,968
Anti-CMV Ig Therapy	58 (4%)	73 (6%)	479 (20%)	610 (12%)	4,968
*Listing Status*					4,963
Status 1	1,193 (90%)	1,130 (90%)	2,149 (90%)	4,472 (90%)	
Status 2	134 (10%)	117 (9%)	240 (10%)	491 (10%)	
Congenital Heart Disease	632 (48%)	525 (42%)	958 (40%)	2,115 (43%)	4,968
Cardiothoracic Surgery	385 (29%)	307 (25%)	673 (28%)	1,365 (27%)	4,968
Pre-Transplant Dialysis	21 (2%)	41 (3%)	64 (3%)	126 (3%)	4,943
ECMO	59 (4%)	102 (8%)	116 (5%)	277 (6%)	4,968
Donor Characteristics					
*Age*					4,968
<1 years	317 (24%)	315 (25%)	424 (18%)	1,056 (21%)	
1–3 years	252 (19%)	173 (14%)	347 (15%)	772 (16%)	
3–6 years	148 (11%)	111 (9%)	199 (8%)	458 (9%)	
6–12 years	201 (15%)	172 (14%)	317 (13%)	690 (14%)	
>12 years	409 (31%)	478 (38%)	1,105 (46%)	1,992 (40%)	
Gender (Male)	799 (60%)	738 (59%)	1,381 (58%)	2,918 (59%)	4,968
*Race*					4,965
White	1,019 (77%)	936 (75%)	1,832 (77%)	3,787 (76%)	
Black	270 (20%)	277 (22%)	486 (20%)	1,033 (21%)	
Other	36 (3%)	36 (3%)	73 (3%)	145 (3%)	
CMV+ Serology Status	0	864 (69%)	1,857 (78%)	2,721 (55%)	4,968
Diabetes	17 (1%)	4 (0%)	17 (1%)	38 (1%)	4,955
Hypertension	21 (2%)	22 (2%)	55 (2%)	98 (2%)	4,953

**FIGURE 4:**
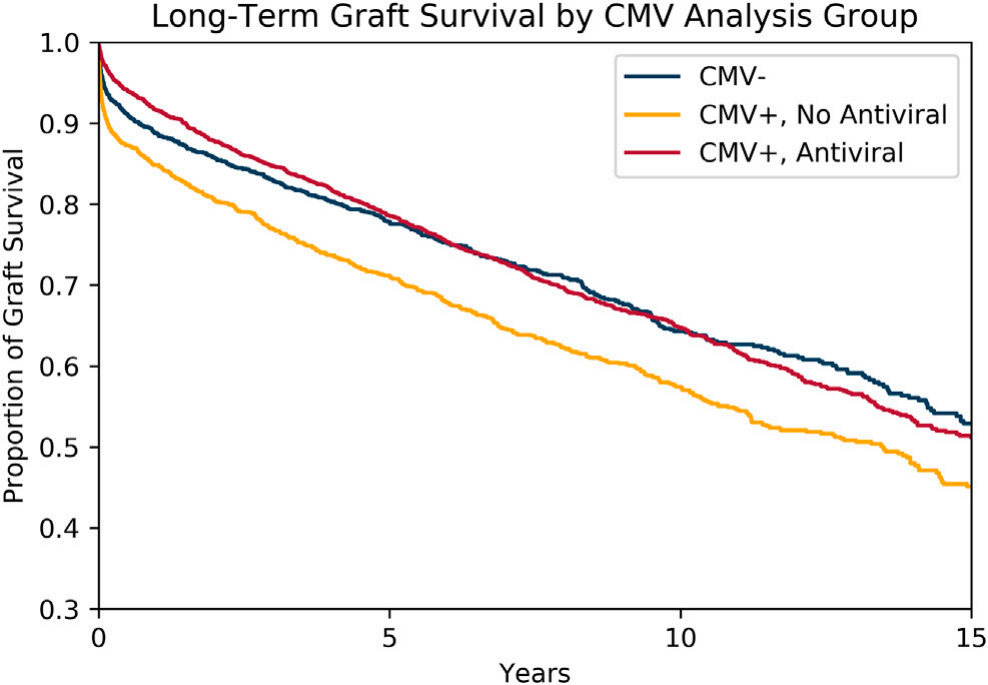
Survival of CMV- Transplants Compared to CMV+ Transplants with and without Antiviral Therapy. Kaplan-Meier survival curves modeling freedom from allograft loss amongst CMV- transplants, CMV+ transplants without antiviral therapy, and CMV+ transplants with antiviral therapy. CMV+ transplants with antiviral therapy demonstrated similar overall graft survival when compared to CMV- transplants.

Kaplan-Meier curves (Figure 4) comparing graft survival across the three groups showed that amongst recipients of CMV+ transplants, those who received antiviral therapy had significantly improved graft survival compared to those who did not (at 10-year 65 vs 57%, log-rank *p*-value < .001). The difference in graft survival between the two groups was observed early after transplantation. Recipients of CMV+ transplants who received antiviral therapy achieved similar rates of long-term graft survival as recipients of CMV- transplants.

In the unadjusted Cox proportional hazards model, when compared to CMV- transplants, CMV positivity without antiviral therapy had a hazard ratio of 1.34 (*p*-value < .001, 95% CI 1.18–1.51). In a fully-adjusted multivariable model, this hazard ratio was 1.21 (*p*-value = 0.003, 95% CI 1.07–1.37). Meanwhile, in the unadjusted model, antiviral use amongst CMV+ transplants had a hazard ratio of .77 (*p*-value < .001, 95% CI 0.69–0.86) when compared to CMV+ transplants that did not receive antiviral therapy. In the fully-adjusted model, this hazard ratio was 0.82 (*p*-value < .001, 95% CI .74–.92). These hazard ratios changed minimally with the addition of an antiviral use propensity score to the model (Table 2).

**TABLE 2:** Hazard ratios for CMV seropositivity and antiviral therapy.

	Unadjusted			Adjusted			Adjusted model with propensity score		
	HR	95% CI	*p*-value	HR	95% CI	*p*-value	HR	95% CI	*p*-value
CMV positivity without antiviral therapy	1.34	1.18–1.51	<0.001	1.21	1.07–1.37	0.003	1.25	1.10–1.42	0.001
Antiviral therapy in CMV+ transplants	0.77	0.69–0.86	<0.001	0.82	0.74–0.92	<0.001	0.82	0.73–0.92	<0.001

Other significant risk factors from the multivariable model included post-transplant dialysis, donor age, donor male gender, recipient congenital heart disease, recipient ECMO, recipient prior cardiothoracic surgery, and SRTR-reported recipient race of Black. Factors associated with improved allograft survival included later transplant year, recipient male gender, higher donor-recipient weight ratio, and donor history of hypertension. Anti-CMV immunoglobulin was not statistically significantly associated with graft survival. The complete results of the fully-adjusted multivariable model are summarized in Supplementary Table 1.

As noted in the Kaplan-Meier survival curves, the difference in graft survival between the three groups was observed early after transplantation. Furthermore, across the entire observation period, the test for deviation from proportional hazards was highly significant (*p*-value < .001).

Therefore, subsequent analysis focused on the first year after transplantation. Within that time period, the proportion of graft loss was 11% in CMV- transplants, 15% in CMV+ transplants without antiviral therapy, and 8.4% in CMV+ transplants with antiviral therapy. This translates to an absolute difference in graft loss within the first year of +4% for CMV seropositivity without antiviral use (compared to CMV negativity) and −6.6% for antiviral use in CMV+ transplants.

In the unadjusted Cox proportional hazards model estimating graft loss within the first year, when compared to CMV- transplants, CMV positivity without antiviral use had a hazard ratio of 1.40 (*p*-value = .002, 95% CI 1.13–1.74). Antiviral use amongst CMV+ transplants had a hazard ratio of 0.52 (*p*-value < .001, 95% CI .43–.64). In the fully-adjusted model, these hazard ratios were 1.32 (*p*-value = .014, 95% CI 1.06–1.64) and .59 (*p*-value < .001, 95% CI 0.48–0.73), respectively. These hazard ratios changed minimally with the addition of an antiviral use propensity score to the model (Table 3). In this model, factors significantly associated with graft loss included post-transplant dialysis, recipient congenital heart disease, recipient history of cardiothoracic surgery, recipient ECMO, and SRTR-reported recipient race of Black. Factors associated with improved graft survival included later transplant year, recipient age, and recipient male gender. Complete results of this fully-adjusted multivariable model are summarized in Supplementary Table 2. Finally, when excluding recipients who experienced graft loss within the first week of transplant from the model, the hazard ratios remained significant at 1.33 (*p*-value = .024, 95% CI 1.04–1.71) and .68 (*p*-value < 0.001, 95% CI 0.54–0.85), respectively.

**TABLE 3:** Hazard ratios of graft loss within the first year after transplantation for CMV seropositivity and antiviral therapy.

	Unadjusted			Adjusted			Adjusted model with propensity score		
	HR	95% CI	*p*-value	HR	95% CI	*p*-value	HR	95% CI	*p*-value
CMV positivity without antiviral therapy	1.40	1.13–1.74	0.002	1.32	1.06–1.64	0.014	1.39	1.10–1.74	0.005
Antiviral therapy in CMV+ transplants	0.52	0.43–0.64	<0.001	0.59	0.48–0.73	<0.001	0.61	0.49–0.75	<0.001

## Discussion

This longitudinal cohort study of a large, national database of pediatric heart transplants demonstrates that CMV seropositivity (recipient or donor) is associated with decreased graft survival time in recipients who did not receive CMV antiviral therapy after transplant. Furthermore, it demonstrates that the use of CMV antiviral medication with either ganciclovir or valganciclovir in CMV seropositive transplants is associated with a significant improvement in graft survival. This relationship is observed early after transplant.

CMV is a herpesvirus that leads to persistent latent infection after resolution of acute illness. History of CMV infection is common in the general population, and in immunocompetent hosts is usually clinically insignificant (19). However, CMV infection can cause serious morbidity in immunocompromised persons and is of particular importance in transplant recipients. Acute illness can occur through first-time exposure to the virus or reactivation of latent infection. There is also growing evidence that in addition to acute illness, CMV also contributes to graft loss in transplant recipients through longer-term effects such as the development of cardiac allograft vasculopathy (2–6).

Traditionally, the risk for acute CMV infection is stratified by donor (D) and recipient (R) serostatus combination, with D+/R- considered to be the highest risk. Thus, these patients are more likely to receive anti-CMV medication. Although this pattern of risk has been observed in several studies (9,20), other studies have casted doubt on this conventional wisdom. For example, one transplant center observed that D+/R+ transplants actually had the highest risk for CMV infection in their prospective cohort of pediatric heart transplant recipients (21).

There have been only a few attempts at estimating the relationship between CMV seropositivity and CMV antiviral therapy on graft survival. Such studies in pediatric transplantation have yielded conflicting results. For example, a study of pediatric heart transplant recipients at a single institution by Hussain *et al.* found that recipient CMV seropositivity was significantly associated with the development of cardiac allograft vasculopathy and decreased graft survival (22). In this cohort, CMV antiviral use was too infrequent to adequately analyze. On the other hand, analysis of an earlier version of the SRTR database by Snydman *et al.* demonstrated a positive association between CMV antiviral therapy and graft survival (13). However, this study was unable to demonstrate a statistically significant association between CMV serostatus and graft survival.

Meanwhile, a large study of the Pediatric Heart Transplant Society (PHTS) database by Mahle *et al.* failed to show any association between CMV serostatus and graft survival or between CMV antiviral therapy and survival (9), and an analysis of pediatric recipients in the Registry of the International Society for Heart and Lung Transplantation (ISHLT) also found no association between donor-recipient CMV serology mismatch and 1-year mortality (23).

One explanation for such inconsistent results is that the use of antiviral therapy is naturally associated with CMV seropositivity. Therefore, it is possible that a potentially detrimental effect of CMV seropositivity and a potentially favorable effect of CMV antiviral therapy may negatively confound each other, making the true underlying impact of these exposures difficult to detect. This is the reason for our three-group analysis, which allows one to statistically quantify the relationship between CMV seropositivity (without antiviral treatment) and graft survival as well as the relationship between antiviral therapy amongst CMV+ transplants and graft survival. Furthermore, considering all CMV+ transplants together, regardless of whether the recipient or the donor is positive, also reflects the growing practice of treating all donor or recipient seropositive transplants with CMV prophylaxis (24,25).

Our analysis reveals that CMV serostatus positivity without antiviral therapy has a significant association with decreased graft survival when compared to CMV seronegative transplants. The separation in the survival curves between the groups is observed early after transplant. An adjusted model estimating the risk of graft loss in the first year after transplant shows that CMV positivity without antiviral therapy has a hazard ratio of 1.32 when compared to CMV- transplants.

The hazard ratio of graft loss during that same time period for antiviral therapy amongst CMV+ transplants was .59. Meanwhile, the unadjusted absolute difference in graft loss between treated and untreated CMV seropositive transplants was −6.6% after 1 year, a substantial difference for the field of pediatric heart transplantation.

These findings seem to indicate that CMV serostatus positivity in either the donor or recipient is a significant risk factor for post-transplant graft loss, with a survival difference that is observed unexpectedly early after transplantation. These are important and novel observations from this multicenter cohort study of pediatric heart transplant recipients. Perhaps more importantly, this study also demonstrates that the risk of CMV serostatus positivity appears to be mitigated by antiviral therapy.

Additional subgroup analysis of all four donor-recipient CMV serostatus combinations showed the largest effect of antiviral therapy was observed in donor seropositive transplants (for both seropositive and seronegative recipients). These findings suggest that recipient CMV serostatus positivity may not be as protective as previously believed. Altogether this evidence supports the more widespread use of CMV prophylaxis beyond the traditionally high-risk D+/R- mismatched transplants.

Although a cohort study cannot determine the mechanism underlying the observed relationships, one theory to explain both the magnitude and early timing of graft loss is that a cardiovascular-tropic virus such as CMV may promote early graft failure in the setting of procurement injury and intense immunosuppression. For example, latent CMV infection residing in the graft or recipient endothelium may potentiate procurement and reperfusion injury leading to additional ischemia, graft dysfunction, or rejection in the already pro-inflammatory post-transplant state. Regardless, the results of this study support the need for future investigation into the biological mechanisms of CMV-mediated graft loss and additional studies aimed at the optimization of post-transplantation antiviral regimens.

This registry-based cohort study has inherent limitations. Importantly, the details of dosing, timing, and duration of post-transplant CMV prophylaxis, which varies between centers, is not captured by the binary fields of the SRTR registry. There is also no data on CMV viral load to assess for viremia. Furthermore, the database contains some fields with incomplete data and the questionnaire-based data submission process itself can be prone to errors or oversimplification of clinical details. Another important limitation is that due to incomplete cause of death data in this registry, we were unable to further investigate the relationship between CMV serostatus, antiviral therapy, and specific causes of graft loss, such as rejection, infection, primary graft failure, multiorgan failure, or cardiac allograft vasculopathy, which could have provided more information as to the etiology of CMV-associated morbidity. The interpretation of CMV serology status also has its own limitations. CMV serology status may be falsely positive from exposure to blood products, which are commonly used in heart failure patients. Infant serology status is also limited by the possibility of positivity from passively-acquired maternal antibodies (15).

Finally, like all observational studies, there may be unmeasured confounders that could explain the observed associations. However, thorough analysis was done to address potential sources of bias by considering demographic information, era (through inclusion of transplant year), and conventional clinical characteristics in our models. Multivariable models were also adjusted by a propensity score estimating the use of antiviral medication as well as the occurrence of post-transplant dialysis, since renal failure may delay or limit the use of antiviral medication. In order to minimize potential selection bias (e.g., the antiviral use variable may be inadvertently selecting those recipients who survived long enough to receive treatment) an additional model was calculated excluding those recipients with allograft loss within the first week after transplant. This analysis demonstrated that these additional factors had little effect on the strength of the association between CMV serostatus, CMV antiviral therapy, and risk of graft loss.

## Conclusion

This large-scale analysis of a multi-institutional national database of pediatric heart transplant recipients demonstrates that CMV serostatus positivity, as defined by either donor or recipient positivity, is associated with an increased risk of graft loss that is largely observed early after transplantation in recipients who are not treated with CMV antiviral therapy. Additionally, this study shows that the use of CMV antiviral therapy amongst CMV seropositive transplants is associated with a significant improvement in graft survival. When treated with CMV antiviral therapy, recipients of CMV seropositive transplants experienced similar graft survival times as recipients of seronegative transplants. These findings suggest that patients involved in a CMV serostatus positive transplant with either the donor *or* recipient being CMV+ may benefit from CMV antiviral medication after transplantation. Of course it is important to recognize that these findings are limited by the observational and registry-based nature of the study and do not embody all of the complexity of the medical management of heart transplant patients. However, this serves as strong motivation for future studies into the mechanisms behind CMV-mediated allograft loss and prospective studies aimed at optimizing post-transplant antiviral regimens.

## Data Availability

Publicly available datasets were analyzed in this study. This data can be found here: https://www.srtr.org/about-the-data/the-srtr-database.

## Abbreviations

CI, Confidence Interval; CMV, Cytomegalovirus; ECMO, Extracorporeal Membrane Oxygenation; SRTR, Scientific Registry of Transplant Recipients; SD, Standard Deviation; IQR, Interquartile Range.
