# Supplementary material for: Association Between Cytomegalovirus Serostatus, Antiviral Therapy, and Allograft Survival in Pediatric Heart Transplantation
Source: Transpl Int. 2022 Mar 16;35:10121. doi: 10.3389/ti.2022.10121 (PMC8964945; doi:10.3389/ti.2022.10121)
Supplement: Supplementary file 1 [file DataSheet1.docx]

**Supplemental Appendix**

The complete results from the fully-adjusted multivariable Cox proportional hazards models are presented in the following tables. Supplementary Table 1 summarizes the significant risk factors from the multivariable Cox proportional hazards model estimating graft loss over the entire observation period. Supplementary Table 2 summarizes the significant risk factors from the multivariable Cox proportional hazards model estimating graft loss during the first year after transplantation.

**Supplementary Table 1. Complete Results from Long-Term Multivariable Cox Proportional Hazards Model.**

|  |  | Hazard Ratio | 95% CI | *p*-value |
| --- | --- | --- | --- | --- |
| CMV+ Without Antiviral vs. CMV- | | 1.25 | 1.10 – 1.42 | 0.001 |
| CMV+ With Antiviral vs. CMV+ Without | | 0.82 | 0.73 – 0.91 | <0.001 |
| Antiviral Use Propensity Score | | 0.92 | 0.26 – 3.23 | 0.902 |
| Transplant Year | | 0.95 | 0.93 – 0.97 | < 0.001 |
| Post-Transplant Dialysis | | 3.21 | 2.75 - 3.75 | < 0.001 |
| Donor Age | | 1.02 | 1.01 - 1.02 | < 0.001 |
| Donor Male Gender | | 1.18 | 1.07 - 1.29 | 0.001 |
| Donor Hypertension | | 0.60 | 0.38 - 0.95 | 0.028 |
| Recipient Male Gender | | 0.84 | 0.76 - 0.92 | < 0.001 |
| Recipient Congenital Heart Disease | | 1.41 | 1.27 - 1.56 | < 0.001 |
| Recipient Prior Cardiothoracic Surgery | | 1.17 | 1.03 – 1.32 | 0.015 |
| Recipient ECMO | | 1.68 | 1.33 - 2.13 | < 0.001 |
| Donor-Recipient Weight Ratio | | 0.86 | 0.77 - 0.95 | 0.004 |
| *Recipient SRTR-Reported Race (Reference: White)* | |  |  |  |
|  | SRTR-Reported Race Black | 1.81 | 1.62 – 2.01 | 0.004 |
|  | SRTR-Reported Race Other | 0.96 | 0.78 – 1.18 | 0.703 |
|  |  |  |  |  |

**Supplementary Table 2. Complete Results from Multivariable Cox Proportional Hazards Model Estimating Graft Loss within the First Year after Transplantation.**

|  |  | Hazard Ratio | 95% CI | *p*-value |
| --- | --- | --- | --- | --- |
| CMV+ Without Antiviral vs. CMV- | | 1.39 | 1.10 – 1.74 | 0.005 |
| CMV+ With Antiviral vs. CMV+ Without | | 0.61 | 0.49 – 0.75 | < 0.001 |
| Antiviral Use Propensity Score | | 2.02 | 0.37 – 11.1 | 0.418 |
| Transplant Year | | 0.93 | 0.91 – 0.96 | < 0.001 |
| Post-Transplant Dialysis | | 7.91 | 6.46 – 9.68 | < 0.001 |
| Recipient Age | | 0.98 | 0.97 – 1.00 | 0.022 |
| Recipient Male Gender | | 0.82 | 0.69 – 0.98 | 0.029 |
| Recipient Congenital Heart Disease | | 2.15 | 1.76 – 2.62 | < 0.001 |
| Recipient Prior Cardiothoracic Surgery | | 1.27 | 1.03 – 1.57 | 0.028 |
| Recipient ECMO | | 1.93 | 1.38 – 2.70 | < 0.001 |
| *Recipient SRTR-Reported Race (Reference: White)* | |  |  |  |
|  | SRTR-Reported Race Black | 1.54 | 1.25 – 1.90 | < 0.001 |
|  | SRTR-Reported Race Other | 1.35 | 0.95 – 1.91 | 0.094 |
